# Supplementary material for: A Vicious NGF-p75NTR Positive Feedback Loop Exacerbates the Toxic Effects of Oxidative Damage in the Human Retinal Epithelial Cell Line ARPE-19
Source: Int J Mol Sci. 2023 Nov 12;24(22):16237. doi: 10.3390/ijms242216237 (PMC10671591; doi:10.3390/ijms242216237)
Supplement: Supplementary file 1 [file ijms-24-16237-s001.zip › ijms-2659493-supplementary.pptx]

## Slide 1
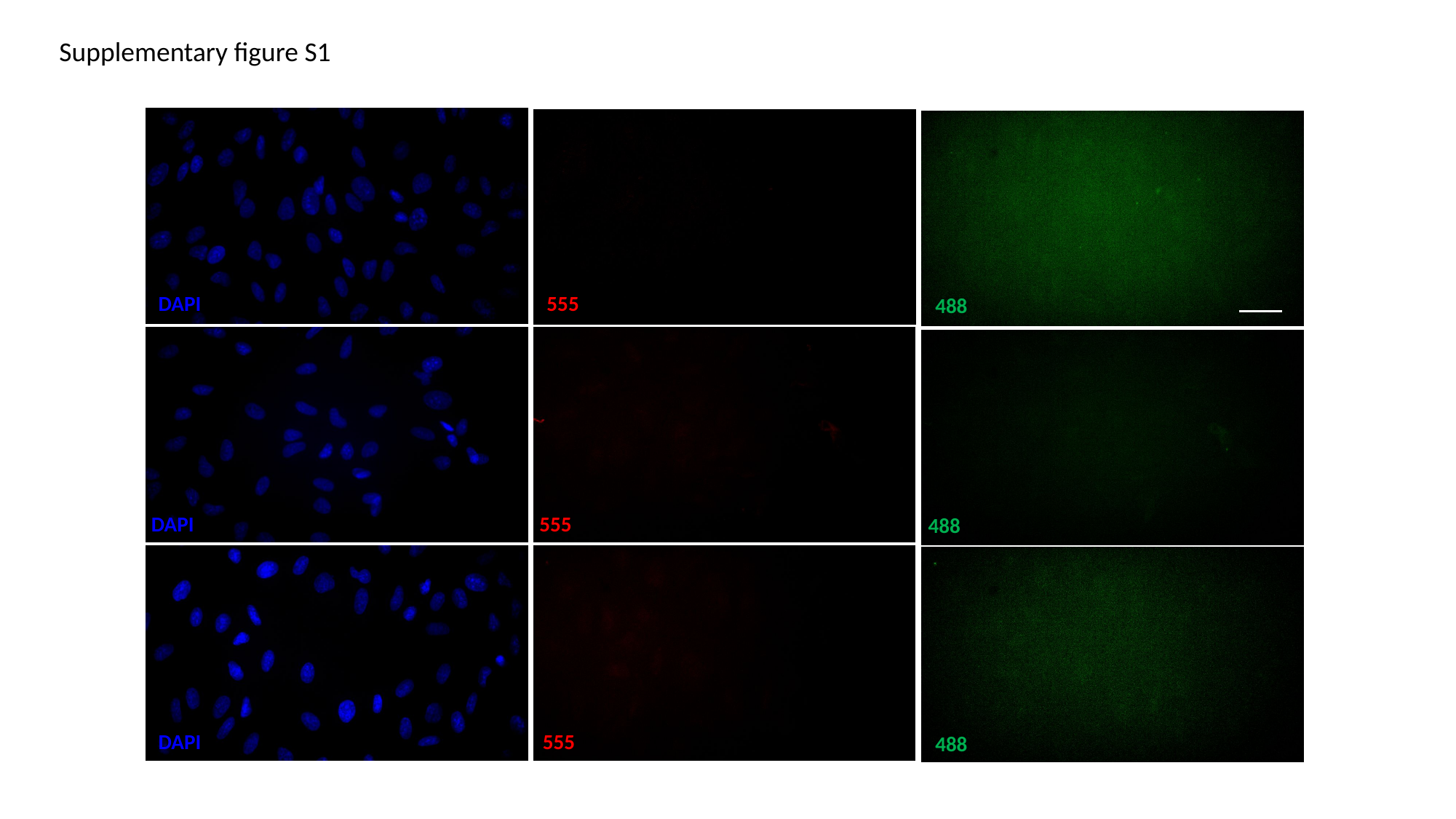

Supplementary figure S1
DAPI
555
488
DAPI
555
488
DAPI
555
488

## Slide 2
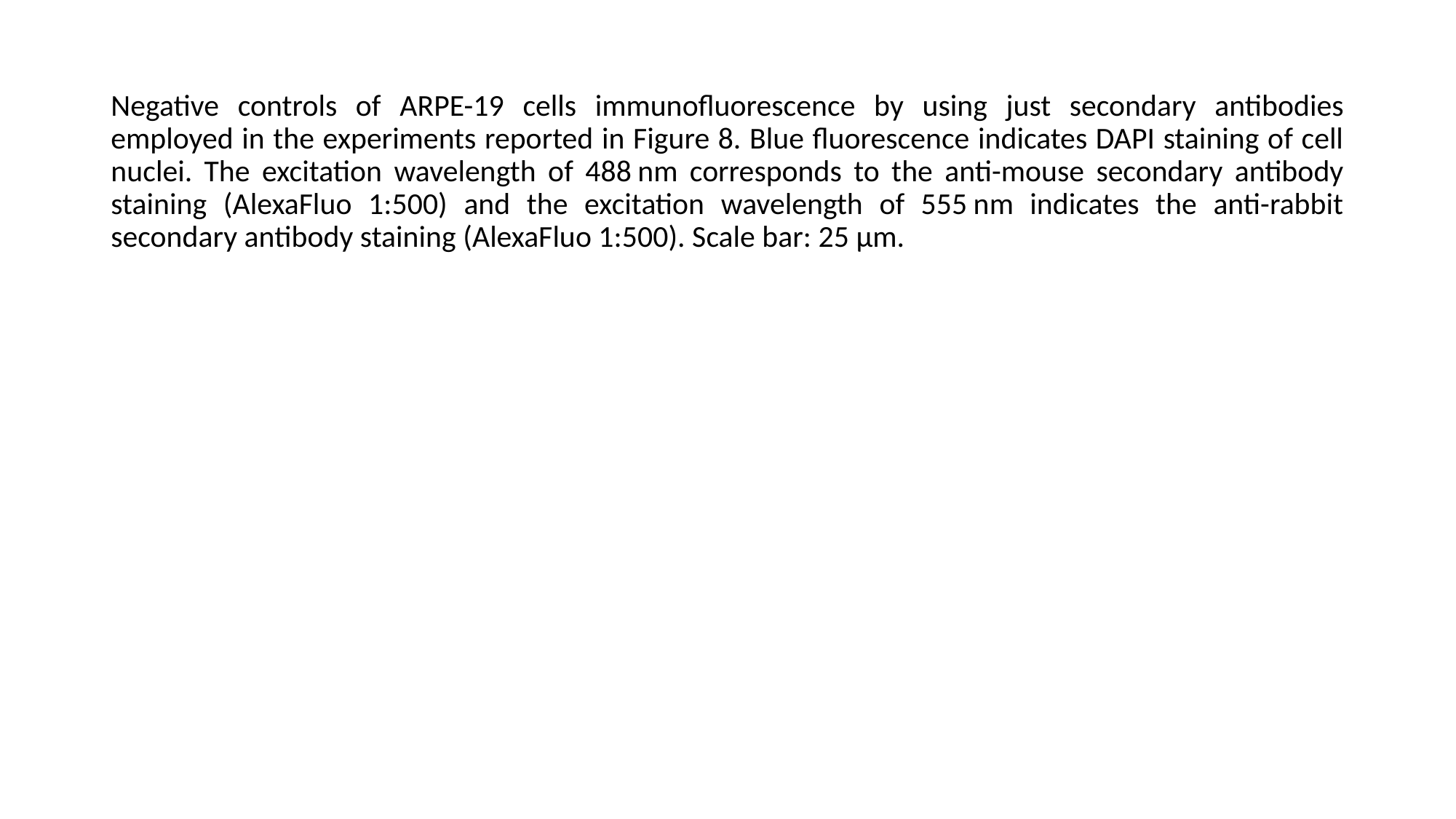

# Negative controls of ARPE-19 cells immunofluorescence by using just secondary antibodies employed in the experiments reported in Figure 8. Blue fluorescence indicates DAPI staining of cell nuclei. The excitation wavelength of 488 nm corresponds to the anti-mouse secondary antibody staining (AlexaFluo 1:500) and the excitation wavelength of 555 nm indicates the anti-rabbit secondary antibody staining (AlexaFluo 1:500). Scale bar: 25 μm.
